# The Evolving Genotypic Profile of HIV-1 Mutations Related to Antiretroviral Treatment in the North Region of Brazil

**DOI:** 10.1155/2015/738528

**Published:** 2015-10-12

**Authors:** Carmen Andréa F. Lopes, Marcelo A. Soares, Diego R. Falci, Eduardo Sprinz

**Affiliations:** ^1^Medical Sciences Post-Graduate Program, Federal University of Rio Grande do Sul, 90035-903 Porto Alegre, RS, Brazil; ^2^Departamento de Genética, Universidade Federal do Rio de Janeiro, 21941-901 Rio de Janeiro, RJ, Brazil; ^3^Programa de Genética, Instituto Nacional de Câncer, 21941-901 Rio de Janeiro, RJ, Brazil; ^4^Infectious Diseases Service, Hospital de Clínicas de Porto Alegre, Universidade Federal do Rio Grande do Sul, 90035-903 Porto Alegre, RS, Brazil

## Abstract

HIV related mutations can be associated with decreased susceptibility to antiretrovirals and treatment failures. There is scarce information about HIV mutations in persons failing HIV treatment in North of Brazil. Our aim was to evaluate evolution of HIV subtypes and mutations patterns related to antiretroviral therapy in this region. We investigated HIV resistance profile in adults failing antiretroviral regimen in Northern Brazil from January, 2004, through December, 2013. Genotype data was evaluated through Stanford University algorithm. There were 377 genotypes from different individuals to evaluate. Resistance mutations were similar to worldwide reports and related to antiretroviral exposure. Most prevalent mutations in the reverse transcriptase gene were M184V (80.1%) and K130N (40.6%). Thymidine associated mutations were more frequent in multiexperienced patients. Most common protease mutations were M46I, V82A, I54V, L90M, I84V, M46L, and L76V. Subtype B was the most prevalent (90.7%). There were differences between subtypes B and non-B mutations. We documented for the first time subtypes and patterns of HIV associated mutations in Northern Brazil. A1 subtype was identified for the first time in this area. Depending on drug regimen and how experienced the patient is, an empirical switch of a failing antiretroviral treatment could be a reasonable option.

## 1. Introduction

The use of highly active antiretroviral therapy (HAART) has dramatically changed the natural course of HIV infection in little more than 30 years of its existence [1, 2]. Although potentially fatal, HIV infection is now considered a chronic and treatable infection [3–6]. Nevertheless, the success of the treatment is closely related to the continuous use of drugs in order to assure persistent plasma viral load (VL) suppression [7, 8].

As HAART cannot eradicate HIV infection, drugs should be friendly to the patient, easy to take, and with reduced or ideally without side effects [9]. Therefore, treatment success is directly related to adherence to treatment without viral replication in plasma [10]. Incomplete HAART viral suppression (due to adherence problems or low potency of the combined medications) is related to the development of viral failure [11]. The continuous replication of HIV under the selective pressure of antiretrovirals (ARVs) will eventually lead to the selection of HIV mutations associated with resistance [12]. The accumulation of mutations further compromises HIV treatment and limits future options if cross-resistance to other ARVs is developed [13, 14]. Therefore, early detection of viral failure is extremely important. The persistence or rebound to detectable levels of HIV in plasma could be an early sign of low adherence or the existence of resistance [15]. The longer the patient is exposed to such treatment, the higher will be the chance to accumulate mutations and develop resistance [16].

An important tool to identify HIV drug resistance mutations is the genotyping test [14]. Although specific patterns of HIV resistance are well established according to the ARV used, they can vary depending on the HIV genetic form (subtype or circulating recombinant form) and drugs used [17, 18].

There are no more than a few Brazilian reports on acquired drug resistance [19–22], transmitted drug resistance, and characterization of the HIV-1 genetic variability [23–27]. HIV-1 subtype B is prevalent throughout the country [19–22], whereas subtypes C and F and their recombinants are relevant in the South and Southeast regions, respectively [28–32]. However, there is scarce data on the circulation of subtypes in the North region of Brazil [33, 34]. The objective of this study was to evaluate the emergence of HIV drug resistance, the possibility of an empirical antiretroviral switch (while waiting for genotyping result), and patterns associated with circulating subtypes and ARVs utilized in a population in Pará, one of the largest states in the North region of Brazil.

## 2. Population and Methods

This was a cross-sectional retrospective study that evaluated HIV genotyping results from individuals with virologic failure (VL greater than 5,000 copies/mL until 2007, and greater than 2,000 copies/mL after 2008), followed up at four public specialized HIV clinics in Belém city, the capital of Pará state, the most populous state of the North of Brazil. Data was collected from January 2004 through December 2013, from the standardized Brazilian HIV genotyping national network of the Ministry of Health (RENAGENO), (http://www.aids.gov.br/pagina/2010/sistema-e-informacao-para-rede-de-genotipagem-sisgeno). The HIV genotyping assay conducted was the ViroSeq HIV-1 Genotype System from Celera Diagnostics (Alameda, CA, USA) in the period from 2004 to 2008 and the TRUGENE System (Siemens, Munich, Germany) from 2009 to 2013. Patients above 18 years of age were enrolled in the study. Only the first available exam was included in those with more than one HIV genotyping text performed.

From the total 464 exams retrieved, 87 were excluded for the following reasons: 19 were conducted in non-RENAGENO laboratories, 12 were from patients under 18 years old, 49 were duplicated, and seven presented incomplete or unreadable results. Therefore, 377 cases remained available for evaluation in the study.

Mutations were described according to the Stanford University HIV Drug Resistance Database (http://hivdb.stanford.edu/). Genotypic resistance was defined as the presence of one or more resistance-related mutations as specified by this database and was further classified according to the ARV class to which they confer reduced susceptibility: nucleoside reverse transcriptase inhibitors (NRTIs), nonnucleoside reverse transcriptase inhibitors (NNRTIs), and protease inhibitors (PIs).

Clinical cases were classified according to the number of regimen failures as follows: first failure, second failure (two treatments), and multifailure (when three or more treatments were experimented prior to genotyping). We also analyzed the association between the mutations observed and the associated HIV-1 subtype (B versus non-B).

The sample size was estimated with the statistical package Winpepi (v.11.37). A prevalence of 82% for subtype B was considered according to Cavalcante et al. [20], with a standard error of 4% and a confidence level of 95%, estimating a minimum size of 355 genotypes for the study. Qualitative variables were evaluated by the Pearson's Chi-square Test, Fisher's Exact Test, or Monte Carlo Exact Significance (when the other tests were not appropriate to be used), and the level of significance was established to 0.05. Data were analyzed in SPSS v.18.0.

The protocol of the study was elaborated according to the resolution number 466 of December 12, 2012, from Conselho Nacional de Saúde (National Health Council) for scientific research in humans and was approved by Comitê de Ética em Pesquisa do Núcleo de Medicina Tropical de Belém do Pará (Ethics Committee in Research of the Nucleus of Tropical Medicine of Belém, Pará) under the number 212.966.

## 3. Results

Overall, 377 genotyping tests were analyzed. The mean age of the patients was 40.7 years (±9.43) and most samples were from men (*n* = 250; 66.3%). The main characteristics of the population in which the tests have been performed can be seen in Table 1. There were 332 (88.1%), 247 (65.5%), and 164 (43.5%) mutations associated with resistance to NRTIs, NNRTIs, and PIs, respectively. In 18 cases (4.8%) no mutation associated with resistance was identified.

Considering HIV subtype, the vast majority of cases were B (*n* = 301; 90.7%), and amongst the non-B, F1 was the most frequent (19 cases; 5.7%). We were able to identify subtype A1 (first case in that region) in the sample. There was no significant difference in the proportion of failures between HIV subtypes during the years studied.

**Figure 1:**
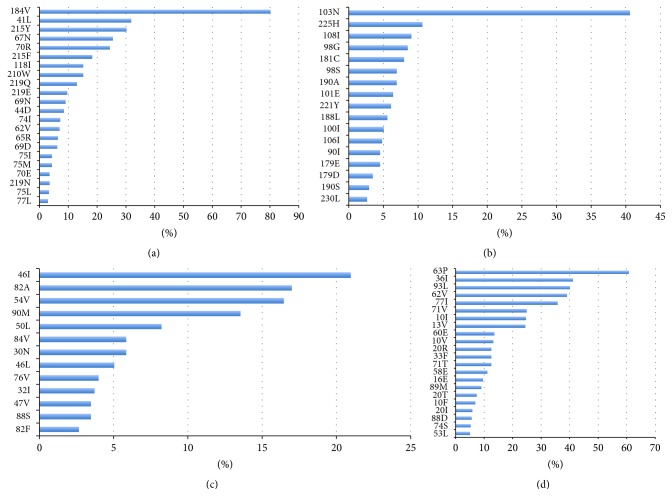
Prevalence of resistance mutations to the NRTIs (a) and NNRTIs (b). Major mutations (c) and accessory mutations related resistance to the PI (d). *N* = 377.

All patients were taking NRTIs at the time the genotyping test was done (Table 1). The most prevalent mutations related to NRTIs were M184V (80.1%), followed by M41L (31.8%), T215Y (30.2%), D67N (25.5%), K70R (24.4%), T215F (18.3%), L210W (15.1%), and V118I (15.1%) (Figure 1(a)). Taking into account multiple mutations together, the thymidine analog mutations (TAM) pathway 1 (including M41L, L210W, and T215Y) was selected in 40.8% of the patients, whereas TAM-2 (including D67N, K70R, T215F, and 58 K219Q/E) was present in 42.2%. The M41L, D67N, V118I, L210W, K219Q, and T69D mutations were more prevalent in experienced patients. The higher the number of ARV regimens already used, the higher the chance of having multiple mutations associated with resistance to zidovudine (ZDV), stavudine (d4T), and tenofovir (TDF) (*p* = 0.011, 0.010, and <0.001, resp.).

Half of the patients (50.1%) were using NNRTIs at the time of genotyping and most of those (83.6%) were on efavirenz (EFV) (Table 1). The K103N mutation (40.6%) and P225H mutation (10.6%) were the most prevalent mutations (Figure 1(b)). At the time of the analysis, K103N (*p* < 0.001) and P225H (*p* = 0.037) were associated with the first regimen failure, while G190S (*p* = 0.013) and M230L (*p* = 0.008) were associated with the second failure. EFV and nevirapine (NVP) had a higher prevalence of resistance in the first failure (*p* < 0.001 for both).

Approximately half of the patients (52.6%) were taking PIs at the moment of the genotyping test (Table 1). The most prevalent major PI mutations were as follows: M46I (21%), V82A (17%), I54V (16.4%), L90M (13.5%), I50L (8.2%), I84V (5.8%), D30N 22 (5.8%), and M46L (5%) (Figure 1(c)). With respect to accessory or secondary mutations, the most frequent were L63P (60.7%), M36I (41.1%), I93L (40.1%), I62V (39%), V77I (35.8%), A71V (24.9%), and L10I (24.7%) (Figure 1(d)). Again, the number of ARV regimens prescribed was positively correlated with the chance of having multiple mutations associated with resistance. In the experienced subgroup, at least seven major (M46I, V82A, I54V, L90M, I84V, M46L, and L76V) and 15 accessory mutations (I62V, A71V, L10I, L10V, K20R, L33F, Q58E, K20T, L10F, T74S, F53L, K43T, G73S, I85V, and A71I) developed and were associated with resistance (*p* < 0.05).

There were some differences regarding the selection of mutations in relation to HIV subtypes (B versus non-B). In the reverse transcriptase gene, the T215F mutation was significantly more selected in non-B subtypes (*p* = 0.023). As for the protease gene, only one major mutation, L76V, was selected more frequently in non-B subtypes (*p* = 0.041). In contrast, several secondary mutations were found to differ between B and non-B subtypes: L63P (*p* < 0.001) and A71T (*p* = 0.021) were higher in subtype B, and M36I (*p* < 0.001), K20R (*p* < 0.001), L10V (*p* = 0.004), L89 M (*p* < 0.001), and F53L (*p* = 0.011) were higher in non-B subtypes.

## 4. Discussion

In the present study we tried to identify, for the first time with a more consistent data, the patterns of HIV resistance according to ARVs used and HIV subtypes in an unexplored geographical region from the North of Brazil. Belém of Pará ranks the eighth Brazilian capital with the highest number of AIDS cases [35]. Although the RENAGENO has started in 2001 and the inclusion of the state of Pará only took place in 2004, with restrictions on the number of tests in the first years, we think our study is representative because it concentrated on 91% of the total genotyping analyses performed in the state.

The resistance-associated mutations profile showed a high prevalence of M184V, TAMs, and K103N in RT. This is in agreement with studies correlating them with the expanded access to HAART in the last decade [8, 36, 37]. Resistance to NRTIs in multifailed patients was related to the presence of the TAMs M41L, D67N, L210W, and K219Q. The accumulation of TAMs, generally over 4, and the TAM-1 mutational pathway confers cross-resistance to nucleoside analogues, including TDF, which may prevent NRTI usage in the composition of subsequent therapeutic rescue regimen [14, 38].

First-generation NNRTIs have low genetic barrier towards drug resistance and for cross resistance in the class [14]. These drugs were part of HAART in 50.1% of the sample (Table 1). The high prevalence of the K103N (40.6%) and the P225H (10.6%) mutations detected was associated with high-level resistance to efavirenz and nevirapine in the first antiretroviral treatment, as was expected [39]. Although our patients did not use the second-generation NNRTI, etravirine (ETR), cross-resistance to this drug was significant in regimens of second failure (31.9%), associated with G190S and M230L mutations. Mutations associated with resistance to ETR have different impact and are usually analyzed by a weighted score of mutations. M230L alone produces moderate resistance to ETR, while G190S requires at least another mutation of equal weight to establish resistance [40]. Fortunately, the prevalence of these mutations was low (<5%), which is in accordance with other studies [41, 42].

Ritonavir-boosted protease inhibitors (PIs) have high genetic barrier towards resistance, requiring a larger number of mutations to present resistance [14, 38]. The group studied here has little experience with PIs and this fact may explain the low prevalence of major PI mutations observed in initial failures. On the other hand, in multifailed individuals, seven major PI mutations associated with resistance were documented: M46I, V82A, I54V, L90M, I84V, M46L, and L76V. Despite the reduced exposure of the studied patients to atazanavir (ATV/r; 21.9%), a large proportion of multifailed patients (56%) displayed genotypic resistance to that drug. Likely, amprenavir/ritonavir (APV/r) was used prior to the genotyping test in only two cases (1%), both multifailed patients, and is not likely to explain the high rate of resistance found (48%) to this drug. Previous PI-based treatments might have contributed to the emergence of PI major mutations M46I, V82A, L90M, I84V, M46L, and L76V, associated with resistance to APV/r.

The selection of the I84V mutation is common to all PIs, including those with the highest genetic barrier, DRV/r and TPV/r [38]. Despite being multifailed patients, DRV/r would be the best PI to rescue HIV treatment, as only 10.9% of the samples demonstrated resistance to the drug. The same was not true with TPV/r, which had documented resistance in 22.1% of the samples. This finding is somehow in accordance with other reports [43]. We think, however, that the Q58E mutation, together with other accessory mutations, L10V, L33F, and K43T, may have contributed to the higher prevalence of TPV/r resistance in relation to DRV/r observed in our study.

HIV-1 subtype B is reported as the predominant viral genetic form in the State of Pará [33], and we corroborate those reports in our study, with a high prevalence of this subtype (90.7%). Indeed, a temporal distribution analysis of B and non-B subtypes during the ten years of the study failed to show significant variation (*p* = 0.280), providing evidence for a stabilized HIV epidemic in terms of diversity in the state of Pará. However, our study reports the presence of subtype A1 for the first time in the state. This subtype is highly prevalent in several African countries and disseminated worldwide [44] but with anecdotal reports in Southeastern Brazil [19]. Non-B subtypes as F1 (5 cases; 1.5%) and C (2 cases; 0.6%) are congruent to the low prevalence reported previously [33].

In our study, L76V PI-associated mutation was the only one that differed significantly in prevalence between B and non-B subtypes, being more frequent in the latter. Specific accessory PI mutations were associated with B and non-B subtypes. L63P and A71T were associated with subtype B, whereas L10V, K20R, M36I, F53L, and L89M were linked to non-B subtypes. Our results are in agreement with previous studies conducted in Brazil that studied the association of PI related polymorphisms with specific HIV genetic forms [19, 22]. There are some data suggesting that primary, compensatory, and polymorphic mutations might intervene with the response and durability of an antiretroviral regimen. Nevertheless, clinical trials are still needed to better define this issue [45–47].

In summary, we found that the prevalence of mutations in the TR of HIV-1 was similar to data already reported. There was a relevant emergence of M184V and TAMs, related to NRTIs, and of K103N, related to the NNRTIs. In PR, mutations M46I/L, I54V, L76V, V82A, I84V, and L90M were associated with PI/r resistance in multifailed patients. The accumulation of NRTIs and PI/r related mutations was associated with resistance in multifailure, confirming the hypothesis that failure to treatment results from the cumulative acquisition of resistance mutations. HIV-1 subtype B was the most prevalent in the state of Pará, and over the decade studied there was evidence of stabilization of this subtype in the state. Based on our findings, we conclude that the mutation patterns of HIV in Pará state were similar to other places worldwide. Also, it is quite reasonable to suppose that, in some situations, depending on drug regimen and how experienced the patient is, an empirical switch of a failing antiretroviral treatment could be a reasonable option while waiting for genotyping result.

## Figures and Tables

**Table 1:** Characteristics of patients failing ARV.

Characteristics	Total (377)
Age in years	
Mean (range)	40.73 (±9.43)
Gender	
Male	250 (66.3%)
CD4+ count (cells/*µ*L)	
Mean (range)	199.93 (±173,36)
HIV RNA level (log_10_ ⁡copies/mL)	
Mean (range)	5.005 (±5,509)
Number of therapeutic failures	
First treatment	91 (24.2%)
Second treatment	85 (22.5%)
Multiexperienced	201 (53.3%)
ARVs before genotyping	
NRTIs + NNRTIs	177 (46.9%)
NRTIs + PI/r	131 (34.8%)
NRTIs + PI	53 (14.1%)
NRTIs + NNRTIs + PI/r	12 (3.2%)
NRTIs + double-boosted PI	2 (0.5%)
NRTIs only	2 (0.5%)

Antiretrovirals, ARV; nucleoside reverse transcriptase inhibitors, NRTIs; nonnucleoside reverse transcriptase inhibitors, NNRTIs; protease inhibitors, PI; boosted protease inhibitors, PI/r.
